# Adsorptive potential of two natural enterosorbents for removing aflatoxin B_1_ under simulated gastric and small intestinal conditions

**DOI:** 10.1007/s12550-025-00588-z

**Published:** 2025-03-20

**Authors:** Tania Karina Vazquez-Ortiz, Lisseth Lozano-Contreras, Ana María Salazar, Monserrat Sordo, Juan de Dios Figueroa-Cárdenas, Alma Vázquez-Durán, Abraham Méndez-Albores

**Affiliations:** 1 Unidad de Investigación Multidisciplinaria L14 (Alimentos, Micotoxinas, y Micotoxicosis), Facultad de Estudios Superiores Cuautitlán (FESC), Universidad Nacional Autónoma de México (UNAM), 54714 Cuautitlán Izcalli, Estado de México Mexico; 2 Instituto de Ciencias Agropecuarias, Universidad Autónoma del Estado de Hidalgo, Av. Universidad Km. 1, 43600 Hidalgo, Mexico; 3 Departamento de Medicina Genómica y Toxicología Ambiental, Instituto de Investigaciones Biomédicas, Universidad Nacional Autónoma de México, 04510 Mexico City, Mexico; 4 CINVESTAV-Unidad Querétaro, Libramiento Norponiente No. 2000, Fraccionamiento Real de Juriquilla, 76230 Querétaro, Mexico; 5 Present Address: Laboratorio de Fisicoquímica L414, FESC, UNAM, 54740 Cuautitlán Izcalli, Estado de México Mexico

**Keywords:** Aflatoxin B_1_, Enterosorbents, Adsorption, Gastrointestinal conditions, Isotherms

## Abstract

A viable strategy for addressing the aflatoxin issue using two enterosorbents prepared from marigold petals and guava leaves was validated. The enterosorbents were characterized via Fourier transform infrared spectroscopy (FTIR), field-emission scanning electron microscopy (FESEM), energy dispersive X-ray fluorescence spectroscopy (EDS), and X-ray diffraction (XRD) to obtain information about the surface functional groups, microstructure, multi-elemental composition, degree of crystallinity, and phase analysis. The potential of the enterosorbents in decreasing aflatoxin uptake and bioavailability under simulated gastrointestinal conditions (including the replication of chemical and enzymatic factors) was estimated using the isotherm models of Langmuir, Freundlich, Temkin, and Dubinin-Radushkevich. Under the simulated gastric and intestinal conditions, marigold removed almost all the mycotoxin at doses of 0.25 and 0.125% (w/w); however, guava leaves efficiently adsorbed the toxin when using doses up to 0.5 and 0.25% (w/w), respectively. Equilibrium adsorption data followed preferentially the Freundlich model, the values of the Freundlich constant (*K*_F_) for marigold were 37.3 and 7.1 times higher than those of guava leaves, respectively. Additionally, the *n* value was > 1, indicative that adsorption was mainly dominated by physical mechanisms. Overall, this research provides insights into the practical application of natural enterosorbents offering a promising approach for AFB_1_ removal.

**Supplementary Information:**

The online version contains supplementary material available at 10.1007/s12550-025-00588-z.

## Introduction

Aflatoxins are a related group of highly toxic metabolites synthesized as part of the secondary metabolism of several species of *Aspergillus* section *Flavi, Nidulante,* and *Ochraceorosei* (Jallow et al. 2021). Up to now, 26 types of aflatoxins have been identified, but the four main aflatoxins are denoted as aflatoxin B_1_ (AFB_1_), aflatoxin B_2_ (AFB_2_), aflatoxin G_1_ (AFG_1_), and aflatoxin G_2_ (AFG_2_) (Liao et al. 2020). AFB_1_ — the most significant toxin in this group — is an extensively studied mycotoxin because it is one of the most toxic and carcinogenic substances of natural origin (Ostry et al. 2017). AFB_1_ toxicity relies on its ability to disrupt cellular processes; this toxin binds to guanine bases in DNA leading to GC → TA transversions. Over time, these transversions accumulate, promoting cancer (Smela et al. 2001). AFB_1_ metabolism also produces reactive oxygen species (ROS), which leads to oxidative stress contributing to cell damage, lipid peroxidation, and further DNA impairment (Kensler et al. 2011). Additionally, AFB_1_ compromises the immune system, reducing resistance to infections and increasing vulnerability to other diseases. Consequently, chronic exposure to AFB_1_, particularly through contaminated food, poses significant health risks to humans.

Unfortunately, contamination with aflatoxins can occur at any stage of food production, from pre-harvest to storage, making it a widespread issue in global food supply. Regulatory authorities worldwide have set limits for allowable aflatoxin levels in food to protect consumers from the harmful effects of these toxic substances. For instance, the US Food and Drug Administration has set regulatory limits for total aflatoxins (the sum of B- and G-series). The limit is 20 µg/kg in most foods, and the limit is much stricter for aflatoxin M_1_ in milk, with an allowable level of 0.5 µg AFM_1_/kg. In contrast, in Regulation (EC) No. 1881/2006, aflatoxin levels were set restrictively to 2 and 4 µg/kg for AFB_1_ and total aflatoxins, respectively. To reduce the content of aflatoxins in food, various strategies have been proposed based on physical, chemical, and biological interventions. Physical adsorption is the most practical, safe, and effective approach to mitigate AFB_1_ exposure in real-time within the digestive system, ensuring rapid toxin binding and excretion without altering normal digestive processes or introducing safety concerns associated with chemical or biological methods. Thus, several materials including activated charcoal, clays, yeast cell wall products (Kong et al. 2014), chitosan and cellulosic polymers (Solís-Cruz et al. 2017), betta-glucans (de Lima Schlösser et al. 2024), as well as various agricultural wastes (Nava-Ramírez et al. 2021; Vázquez-Durán et al. 2022) have been used with positive results.

Recently, there has been an increasing demand for edible flowers and leaves worldwide. Since ancient times, edible flowers have been used in the traditional medicine to treat some diseases, and several studies have supported their positive effects on human health due to their rich composition in bioactive compounds such as polyphenols, flavonoids, and anthocyanins (Fernandes et al. 2017). Mexican marigold (*Tagetes erecta* L.) also known as *cempasúchil* is a flower endemic to Mexico and belongs to the Asteraceae family. Mexican marigold is rich in flavonoids, phenolics, terpenoids, and carotenoids being lutein the most attractive bioactive compound, which justifies its medicinal use with antioxidant, anti-inflammatory, and antimicrobial properties (Estrada et al. 2024). On the other hand, guava (*Psidium guajava* L.) is also a traditional medicinal plant native to tropical regions of Central America. Due to their extensive bioactivity profile and pharmacological effects, guava leaves have been used as tea infusion or extracts to treat a variety of health issues due to their rich content of antioxidants, anti-inflammatory, and antimicrobial compounds (Gutiérrez et al. 2008). Moreover, guava leaves are rich in fiber and vitamin C which aid digestion and boost the immune system. Due to the integral benefits that characterize these plant-based materials that are destined to be consumed with the food, more research is required to assess other potential benefits in real digestive environments. Thus, identifying efficient, natural, and safe decontamination strategies can help mitigate AFB_1_ exposure, protecting both human and animal health while supporting sustainable and cost-effective food safety solutions. In this research, we hypothesize that the use of marigold petals and guava leaves as enterosorbents could provide certain advantages during long-term use, since these natural products can have a dual-purpose role, that is, as an AFB_1_ binder and as a food supplement. To the best of our knowledge, there are no studies on the use of marigold petals and guava leaves as adsorbent materials for AFB_1_ uptake; therefore, the aim of this work was to prepare and characterize two natural enterosorbents and investigate their potential to remove AFB_1_ under simulated gastric and small intestinal conditions.

## Materials and methods

### Chemicals

AFB_1_ standard from *Aspergillus flavus* (CAS number: 1162–65-8), pepsin (CAS number 9001–75-6), pancreatin (CAS number 8049–47-6), bile extract (CAS number 8008–63-7), sodium chloride/sodium chloride solution (CAS number 7647–14-5), hydrochloric acid (CAS number 7647–01-0), sodium bicarbonate (CAS number 144–55-8), potassium chloride (CAS number 7447–40-7), calcium chloride (CAS number 10043–52-4), dimethyl sulfoxide (CAS number 67–68-5), HPLC grade methanol (CAS number 67–56-1), 96% ethanol (CAS number 64–17-5), sodium hydroxide (NaOH; 97% purity; CAS number 1310–73-2), and sodium hypochlorite solution (CAS number 7681–52-9) were acquired from Merck KGaA (Darmstadt, Germany).

### Safety precautions

The AFB_1_ molecule is a potent carcinogen; therefore, the standard solution must be handled with care. Nitrile gloves, lab coats, safety goggles, and disposable masks were worn to prevent skin, eye, and respiratory exposure. All AFB_1_-contaminated glassware was soaked in a sodium hypochlorite solution overnight and then exhaustively washed. Wastes were disposed of according to local regulations.

### Unmodified plant-based enterosorbents

Mexican marigold (*Tagetes erecta*) was cultivated in a commercial greenhouse located in Puebla-Mexico. Guava leaves (*Psidium guajava* L.) were harvested from an organic guava plantation (Coatepec, State of Mexico, Mexico). Plants were cultivated following natural farming practices without the use of any chemical herbicides or pesticides. Marigold flowers and guava leaves were harvested manually out early in the morning on a single occasion (October 2023) and transported in an insulated container to the laboratory. Petals and leaves (without any physical injury) were washed with distilled water, oven dried at 40 °C for 48 h (to ensure the preservation of active components and structural stability), and then finely ground with an electric plate-style mill type C-11–1 (Glen Mills Inc. Clifton, NJ, USA). The ground material was passed through a 250 µm sieve and stored at − 20 °C in a laboratory freezer until further characterization.

### Characterization

Information regarding the chemical composition of the enterosorbents was obtained with a Perkin Elmer Frontier NIR/MIR SP8000 spectrophotometer equipped with an attenuated total reflection (ATR) accessory (DuraSamplIR II, Smiths Detection, Warrington, UK). Samples (50 mg) were placed and measured in transmittance mode after pressing them on the ATR crystal using 32 sequential scans in the range of 4000–400 cm^−1^ with a resolution of 4 cm^−1^. The FTIR spectrometer was calibrated using a mid-infrared (MIR) polystyrene traceable reference material (L1365334, Perkin Elmer, Waltham, MA, USA), and a background spectrum was recorded to eliminate atmospheric interferences. The characteristic absorption bands were identified and analyzed using the Spectrum 10.4.2 software. Moreover, the relative concentration of oxygen-containing and aliphatic groups [OH, (CH)_n_, COOR, and CO] was determined from the integrated transmittance band areas at 3717–3017, 3017–2794, 1794–1537, and 1189–937 cm^−1^, respectively. The chosen bands were selected based on their association with key functional groups known to play a role in the adsorption of AFB_1_.

To obtain high-resolution images of the sample’s surface morphology at the microscale and to determine the elemental composition of specific areas, a field-emission scanning electron microscope accessorized with energy dispersive X-ray fluorescence spectroscopy (FESEM, JEOL 7610F, Tokyo, Japan) was used. Samples were mounted on aluminum stubs and subsequently sputter-coated (Denton Vacuum Inc. Desk V HP, Moorestown, NJ, USA) with a thin layer of gold for 3 min to prevent charging under the electron beam. Microscopy was performed at 5000 × in the secondary electron imaging mode with an accelerating voltage of 2 kV at 6-mm effective working distance. Furthermore, the multi-elemental analysis was conducted using a high-performance micro-spot X-ray source (XTrace), and the generated X-ray fluorescence spectrum was measured with the attached silicon-based energy dispersive XFlash® 6/10 detector (Bruker Nano GmbH, Berlin, Germany).

X-ray diffraction (XRD) analysis was performed with a 2100-Rigaku diffractometer (Rigaku Corp. Tokyo, Japan). The operating conditions were CuK_α_ radiation (*λ* = 1.5405 Å), a fixed power source of 30 kV, and a current of 20 mA. Samples were scanned in the range of 5 to 60° Bragg angles in steps of 1°/0.02 s. Furthermore, the degree of crystallinity was calculated according to the recommendations of Nara and Komiya (1983). The upper diffraction peak area and the total diffraction area over the diffraction angle (5 to 60°) were computed by integration using the Origin 8.0 software (OriginLab, Northampton, MA, USA). The ratio of the upper peak area to the total diffraction area was taken as the degree of crystallinity. Samples with similar moisture contents (~ 7%) were utilized to minimize the effect of this factor on the degree of crystallinity.

### Adsorption experiments

#### Simulated gastric and intestinal juices

The solution that mimics the acidic environment of the stomach was prepared by mixing 3.2 g of pepsin from porcine gastric mucosa (≥ 400 units/mg protein) in 1 L of 0.85% saline. The gastric solution was buffered with hydrochloric acid to a pH of 2.5. The solution that simulated the intestinal condition was prepared by mixing 1 g of pancreatin from porcine pancreas (8 × USP specifications) and 3 g of porcine bile extract in 1 L solution containing 6.5 g NaCl, 1.39 g NaHCO_3_, 0.85 g KCl, and 0.22 g CaCl_2_. The pH of the intestinal solution was adjusted to 6.5. The pH was measured with a glass electrode (Conductronic PC-45, Puebla, Mexico).

#### ***Preparation of the aflatoxin B***_***1***_*** (AFB***_***1***_***) stock solution***

A stock solution of the AFB_1_ (200 μg AFB_1_/mL) was prepared by dissolving the mycotoxin in dimethyl sulfoxide. Then, the solution was diluted in the corresponding simulated gastric and intestinal solution to achieve the desired concentration for the adsorption experiments.

#### ***AFB***_***1***_*** removal assay***

Adsorption experiments were conducted using a Solaris 2000-R small incubated and refrigerated benchtop orbital shaker (Thermo Scientific, Marietta, OH, USA) operated at a constant agitation speed (200 rpm). To determine the optimal quantity, five dosages of the enterosorbents (3.125, 6.25, 12.5, 25, and 50 mg) were tested in 10-mL amber flasks containing 5 mL of the simulated solutions (gastric or intestinal) spiked with AFB_1_ at a concentration of 0.5 µg AFB_1_/mL. Flasks were incubated at 37 °C for 60 min under dim light. Afterward, the optimal adsorbent dose for each simulated gastrointestinal solution was selected for isothermal studies. For this purpose, five different mycotoxin concentrations (0.5, 1, 2, 4, and 8 µg AFB_1_/mL) were evaluated under the same incubation conditions. Control samples (without the addition of enterosorbents) were also included in the experimental to confirm the stability of the AFB_1_ molecule in the gastric and intestinal simulated solutions. All adsorption studies were assayed in quintuplicate.

After the completion of the adsorption process, samples were centrifuged (7000 × *g* for 7 min) and the supernatant was filtered using a PTFE membrane syringe filter with a 0.2-µm pore size. The AFB_1_ concentration was estimated in the filtrate using ultra high-performance liquid chromatography with fluorescence detection (UPLC-FLR). Antibody-based immunoaffinity chromatography columns (Afla-B, VICAM Science Technology, Watertown, MA, USA) were used as a clean-up protocol to isolate the toxin. The adsorption capacity (${q}_{e}$) and the adsorption rate were calculated using the following equations:

1 $${q}_{e}=\frac{{(C}_{0}-{C}_{e}) V}{m}$$

2 $$\text{Adsorption rate}=\frac{{(C}_{0}-{C}_{e})}{{C}_{0}}\times 100$$

In the mathematical expressions, ${C}_{0}$ represents the initial AFB_1_ concentration (µg/L), ${C}_{e}$ represents the equilibrium concentration (µg/L), $V$ symbolizes the volume of the solution (L), and $m$ denotes the mass of the enterosorbent (g).

### Adsorption isotherms

Adsorption isotherms are commonly used to describe how a contaminant interacts with a solid surface as a function of its concentration at equilibrium. In this research, the linearized models of Langmuir, Freundlich, Temkin, and Dubinin–Radushkevich (Table 1) were used to estimate the maximum adsorption capacity (${q}_{e}, \text{mg}/\text{g}$) of the enterosorbents under the gastric and intestinal conditions in relation to the concentration of AFB_1_ (${C}_{e}, \text{mg}/\text{L}$). All the model parameters were assessed by the linear least-squares method using the Origin 8.0 software (OriginLab, Northampton, MA, USA).

**Table 1:** Adsorption isotherm models and their linearized forms

Isotherm	Model	Plot
Langmuir	$\frac{{C}_{e}}{{q}_{e}}=\frac{{C}_{e}}{{q}_{m}}+\frac{1}{b{q}_{m}}$	$\frac{{C}_{e}}{{q}_{e}} vs { C}_{e}$
Freundlich	$ln {q}_{e}=ln {k}_{f}+\frac{1}{n} ln {C}_{e}$	$ln {q}_{e} vs ln {C}_{e}$
Temkin	${q}_{e}=\frac{RT}{bT} ln aT+ \frac{RT}{bT} ln{ C}_{e}$	${q}_{e} vs ln{ C}_{e}$
Dubinin–Radushkevich	$ln {q}_{e}=ln {q}_{m}-\beta {\epsilon }^{2}$	$ln {q}_{e} vs {\epsilon }^{2}$

The Dubinin–Radushkevich isotherm model was used to confirm the type of adsorption (physical or chemical). The adsorption energy (E, kJ/mol) was calculated from the Dubinin–Radushkevich parameter β using the following equation:

3 $$E= \frac{1}{\sqrt{2\beta }}$$

### *Analysis of aflatoxin B*_*1*_* (AFB*_*1*_*)*

An ultra-performance liquid chromatography with fluorescence detection (UPLC-FLR) methodology was developed and validated for the determination of AFB_1_ in the filtrates. Briefly, an aliquot (10 µL) of the cleaned-up sample was separated into an ACQUITY UPLC BEH C18 column (2.1 × 100 mm, 1.7 µm) using a Waters ACQUITY H-Class System (Waters, Milford, MA, USA). The AFB_1_ was eluted under isocratic conditions with a mobile phase of HPLC grade water/methanol/acetonitrile (64/18/18) delivered with a flow rate of 400 µL/min by a quaternary solvent manager. The mycotoxin was detected by an UPLC-optimized fluorescence detector (Waters, Milford, MA, USA) using excitation and emission wavelengths of 365 and 435 nm, respectively. The AFB_1_ concentration was calculated using the standard reference with a calibration curve.

### Method validation

The performance of the UPLC-FLR methodology was validated using the guidelines for single-laboratory validation of analytical methods for trace-level concentrations of organic chemicals elaborated by the AOAC/FAO/IAEA/IUPAC (Alder et al. 2000). Four parameters including limit of detection (LOD), limit of quantification (LOQ), recovery, and linearity were estimated. The LOD was determined as the lowest concentration of the toxin that produced a chromatographic peak with a signal-to-noise ratio of 3, and the LOQ was recorded at a signal-to-noise ratio of 10. Different concentrations of AFB_1_ (from 8 to 250 ng AFB_1_/g) were added to blank matrix samples for recovery experiments using the optimized clean-up protocol. The linearity was estimated using standard solutions of AFB_1_ over the range of 10 to 1000 ng AFB_1_/L. In general, LOD and LOQ values were found to be 2.0 and 6.7 ng AFB_1_/L, respectively. The average recoveries ranged between 92 and 97%, and the linearity estimated with the coefficient of determination (*R*^2^) was 0.9984. The results indicated that the developed and validated UPLC-FLR methodology can be applied to the trace analysis of AFB_1_ in the filtrates.

### Experimental design and statistical analysis

The experiment was conducted as a completely randomized design with five replicates. Adsorption data was analyzed by one-way analysis of variance, and the differences between the means were compared using Tukey’s honestly significant difference post hoc test with the Minitab 16.0.1 software (Penn State University, State College, PA, USA). A significance value of *α* = 0.05 was used to reject the null hypothesis.

## Results and discussion

### Characterization

**Fig. 1:**
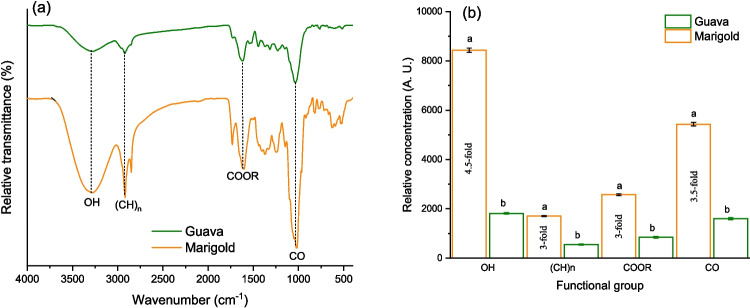
Comparative Fourier transform infrared spectra of the enterosorbents prepared from marigold and guava leaves (**a**) and the relative concentration of their main functional groups (**b**)

The FTIR spectrum of the unmodified enterosorbents after subtracting the background is shown in Fig. 1a. The band assignments followed the work previously published by Zavala-Franco et al. (2018). In general, it can be clearly seen that the main characteristic transmittance bands are similar, demonstrating that both enterosorbents have identical surface functional groups. However, significant differences in the relative transmittance of the main FTIR bands were evident. Consequently, the corresponding infrared spectrum was divided into four regions for the semiquantitative analysis of the functional groups such as hydroxyl (3717–3017 cm^−1^), aliphatic (3017–2794 cm^−1^), carboxyl (1794–1537 cm^−1^), and the C-O bond in carbohydrates (1189–937 cm^−1^). In the enterosorbent prepared from marigold, it was found that the O–H, (CH)_n_, COOR, and C–O bond concentrations were significantly higher than those in the guava leaves (Fig. 1b). Thus, marigold has up to 4.5-fold and threefold increase in O–H and COOR groups which would make its surface more favorable to the adsorption of AFB_1_ molecules (via hydrogen bonding) in the solution that mimics the acidic environment of the stomach. However, in the solution that simulated the intestinal condition, the COOR group is mainly found in its unprotonated form (the pK_a_ of a carboxylic acid is around 5). Thus, in the intestinal environment, the resulting carboxylate ion will not establish hydrogen bonds with the oxygen atoms of the ether, carbonyl, and methoxy groups in the AFB_1_ molecules (Vázquez-Durán et al. 2021). Besides, marigold has also up to threefold increase in aliphatic groups, resulting in a material with a low hydrophilicity surface which is favorable to the adsorption of AFB_1_ via hydrophobic interactions.

**Fig. 2:**
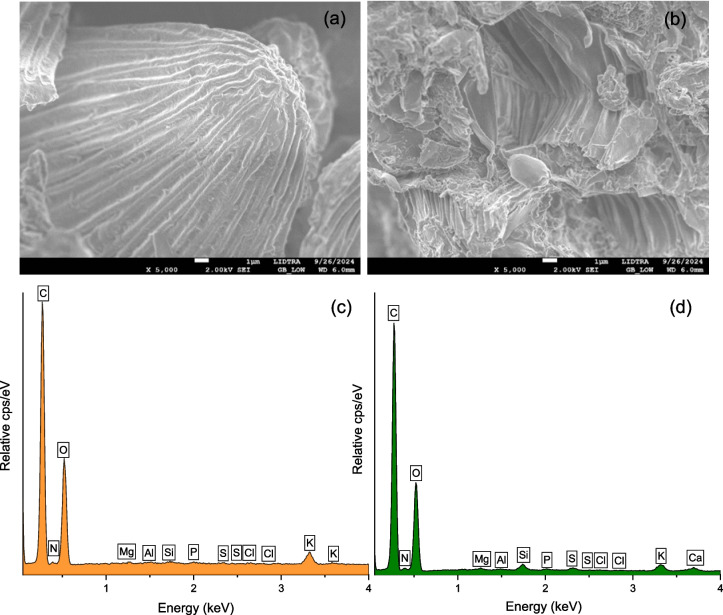
FESEM micrographs of the enterosorbents prepared from marigold and guava leaves (**a**, **b**), and their corresponding energy-dispersive X-ray spectroscopy spectrum (**c**, **d**)

The FESEM micrographs at 5000 × magnification are shown in Fig. 2a and b. As seen, the enterosorbents have heterogeneous surfaces with varying degrees of porosity and roughness, reflecting the complex structure of these plant-based materials. The roughness increases the surface area available for adsorption, making these materials more effective to capture the pollutant. It was also evident from the FESEM images that the enterosorbents showed a sheet-like structure with several interconnected channels that facilitate the movement of the contaminant, where there are too many possibilities for the toxicant to interact with the surface functional groups. These intricate structural features reveal a complex surface texture of both enterosorbents, which contribute to their effectiveness in adsorbing the mycotoxin.

Moreover, the micro-elemental analysis (Fig. 2c and d) revealed insights into the composition of the enterosorbents. In general, the EDX pattern of marigold showed the presence of significant quantities of oxygen compared to those found in guava leaves (42.88 vs 37.91%). Other signals of significant intensities were carbon, silicon, sulfur, chlorine, and potassium. Traces of magnesium, aluminum, phosphorous, and calcium were also observed (Table 2). Although every plant-based adsorbent has unique structural properties and micro-elemental composition, one common feature shared is that they contain different amounts of active surface functional groups. In line with these results, our recent studies confirmed that the oxygen-containing functional groups are crucial for AFB_1_ adsorption (Méndez-Albores et al. 2020; Vázquez-Durán et al. 2023). Thus, in this research, the uptake of the carcinogen may be expected to be higher when using marigold, since this material contains significant amounts of oxygenic functional groups on its surface.

**Table 2:** The micro-elemental composition (Wt %) of the enterosorbents

Element	Enterosorbent	
	Marigold petals	Guava leaves
C	48.04 ± 1.29^a^	52.27 ± 1.07^b^
N	4.69 ± 0.48	5.51 ± 0.45
O	42.88 ± 0.84^a^	37.91 ± 0.39^b^
Mg	0.16 ± 0.01	0.21 ± 0.04
Al	0.16 ± 0.02	0.10 ± 0.01
Si	0.37 ± 0.07^a^	0.72 ± 0.02^b^
P	0.19 ± 0.03	0.19 ± 0.02
S	0.10 ± 0.03^a^	0.40 ± 0.02^b^
Cl	0.16 ± 0.02^a^	0.06 ± 0.01^b^
K	3.24 ± 0.09^a^	1.68 ± 0.01^b^
Ca	ND	0.94 ± 0.02

Mean values ± standard error. Means with a different letter in the same row are statistically different (Tukey *p* < 0.05). ND = Not detected

**Fig. 3:**
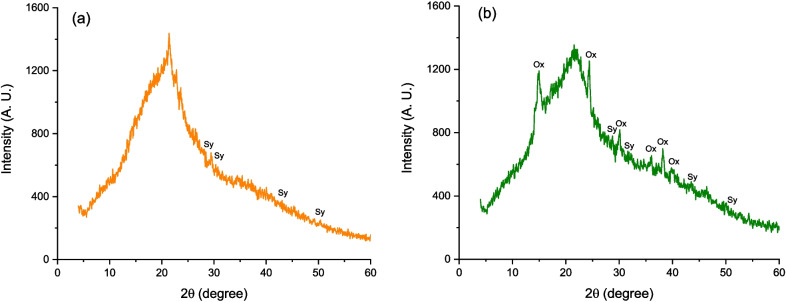
Representative X-ray diffraction patterns of the enterosorbents prepared from marigold (**a**), and guava leaves (**b**). Sy, sylvite; Ox, calcium oxalate

Figure 3 shows the XRD patterns of the enterosorbents. In general, marigold and guava leaves presented both amorphous and crystalline phases. Marigold showed small diffraction peaks at 27.3, 31.6, 45.3, and 56.4° 2*θ*, related to the pure phase of potassium chloride (standard card JCPDS: 004–0587). Moreover, guava leaves had diffraction peaks like those of potassium chloride (sylvite) and crystalline peaks identified as calcium oxalate (standard card JCPDS 014–0768).

Regarding crystallinity, marigold showed the lowest degree of crystallinity (< 1%), whereas guava leaves exhibited a moderate crystallinity value (up to 7.3%) due to the presence of considerable amounts of salts such as potassium chloride and calcium oxalate. The amorphous and crystalline phases in plant-based adsorbents significantly influence their adsorption properties. For instance, amorphous regions lack a well-defined crystalline structure, which often leads to greater surface area and more accessible sites for adsorption. Thus, the lack of order in the amorphous region of marigold exposed more functional groups such as hydroxyl and carboxyl that can interact with the AFB_1_ molecule in the simulated gastrointestinal sections. Moreover, the disorder in the amorphous region could facilitate a faster diffusion of the mycotoxin due to less structural resistance; consequently, amorphous regions contribute more to the overall adsorption capacity, making marigold a more effective enterosorbent in capturing the toxicant.

### Adsorption studies

#### Optimal doses of the enterosorbents

**Fig. 4:**
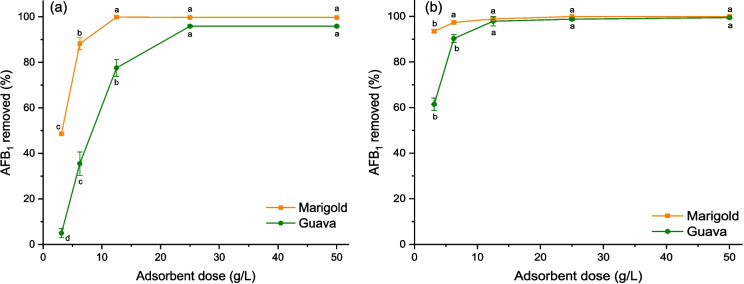
The effect of the enterosorbent dose on the removal rate of AFB_1_ under the conditions of the stomach (**a**) and intestine (**b**). Mean values not sharing a common superscript letter are significantly different (Tukey *p* < 0.05)

The enterosorbents (marigold petals and guava leaves) were evaluated in two different solutions simulating the *in vivo* conditions of the gastrointestinal tract (stomach, pH 2.5; and intestine, pH 6.5). In a preliminary examination, five dosages were tested corresponding to 0.0625, 0.125, 0.25, 0.5, and 1% (w/w). Figure 4 shows the effect of the enterosorbent dosage on the removal of AFB_1_ under the conditions of the stomach and intestine. The results showed that marigold removed almost all the mycotoxin at doses of 0.25 and 0.125% (w/w); however, guava leaves efficiently adsorbed the toxin when using doses up to 0.5 and 0.25% (w/w), respectively. In general, to achieve 97% removal of the toxin under the simulated gastric and small intestinal conditions, a double dose of guava leaves was always required. Therefore, the doses that presented the highest adsorption percentages were used for the following in vitro tests.

#### Adsorbate concentration

Table 3 shows the removal percentage achieved by each enterosorbent at different AFB_1_ initial concentrations (from 0.5 to 8 µg AFB_1_/mL) under the simulated conditions of the gastrointestinal tract. In general, at the acidic pH of the stomach (pH 2.5), and for the five AFB_1_ concentrations tested, marigold presented adsorption rates significantly higher (*p* < 0. 001) than guava leaves, even considering that guava leaves were used at a dose of 0.5% (w/w). However, at the conditions of the intestinal section (pH 6.5), there were no statistical differences (*p* > 0.05) among the tested enterosorbents in the entire range of the AFB_1_ concentration evaluated (Table 3). According to Table 2, both enterosorbents showed the highest AFB_1_ removal efficiencies (above 93.88%), even at the maximum aflatoxin concentration tested (8 µg AFB_1_/mL), suggesting that marigold and guava leaves contain abundant vacant adsorption sites to efficiently remove the toxicant (Nava-Ramírez et al. 2021). The removal efficiency of marigold and guava leaves is significantly higher than those reported for other plant-based adsorbents (Avantaggiato et al. 2014; Shar et al. 2016; Fernandes et al. 2019) and comparable with the efficiency of naturally or chemically modified clays (Zhou 2016; Sun et al. 2018; Zhao et al. 2022).

**Table 3:** Percentage of adsorption of the enterosorbents under the simulated conditions of the gastrointestinal tract

AFB_1_ (µg/mL)	Adsorption (%) at pH 2.5		*p*-value	SEM	Adsorption (%) at pH 6.5		*p*-value	SEM
	Marigold petals	Guava leaves			Marigold petals	Guava leaves		
0.5	99.21^a^	95.07^b^	0.001	2.07	98.74^a^	99.99^a^	0.340	0.63
1	99.29^a^	93.94^b^	0.000	2.68	99.11^a^	99.99^a^	0.251	0.44
2	99.11^a^	94.01^b^	0.000	2.55	97.47^a^	99.00^a^	0.177	0.77
4	99.33^a^	93.51^b^	0.000	2.91	98.46^a^	98.53^a^	0.907	0.05
8	99.51^a^	93.88^b^	0.000	2.82	98.62^a^	97.41^a^	0.128	0.61

^a^^,^^b^Means with a different letter in the same row are statistically different (Tukey *p* < 0.05). *SEM* standard error of the mean

#### Adsorption isotherms

In this work, the experimental data were fitted using the linearized isotherm models of Langmuir, Freundlich, Temkin, and Dubinin–Radushkevich to calculate the maximum adsorption capacity of each enterosorbent under the simulated conditions of the gastrointestinal tract. The adsorption isotherms in the conditions of the stomach and intestine environment are shown in Fig. S1 and Fig. S2, respectively. Additionally, Table 4 shows the results of the isotherm parameters at a temperature of 310 K. In general, in the two sections of the gastrointestinal tract, the Freundlich isotherm was the most adequate model to represent the experimental data, resulting in higher coefficient determination values. However, at the acidic pH of the stomach, the Freundlich model presented higher coefficient determination values (up to 0.997) in comparison to those found at pH 6.5 (up to 0.915). The Freundlich model is particularly useful for describing adsorption in systems where the surface of the adsorbent is heterogeneous with energetically different sites (Freundlich 1924). This might be explained by the surface of the enterosorbents, which is not uniform as seen during FESEM imaging. The empirical Freundlich model also assumes that multilayer adsorption is possible; consequently, in this research, there was no evidence that saturation at high concentrations of the contaminant (up to 8 µg AFB_1_/mL) was reached. Although considerable coefficient determination values were attained when data was fitted with the Dubinin–Radushkevich (from 0.887 to 0.953) and with the Temkin model (from 0.714 to 0.868), the best fit obtained was with the Freundlich isotherm model (Table 4). In this context, Loffredo et al. (2020) evaluated the adsorption of the mycotoxin ochratoxin A (OTA) using novel low-cost biosorbents. Equilibrium adsorption data of OTA onto clementine peel, coconut fiber, and spent coffee grounds followed preferentially the Freundlich model. Moreover, very recently, Zhang et al. (2024) assessed the efficiency of AFB_1_ removal by *A. luchuensis* YZ-1 spores using two commonly adsorption isotherm models, Langmuir and Freundlich. The authors concluded that the Freundlich model better described the adsorption process of AFB_1_ onto the *A. luchuensis* YZ-1 spores. These results are consistent with our findings.

**Table 4:** Isotherm parameters for the adsorption of AFB_1_ onto the enterosorbents under the simulated conditions of the gastrointestinal tract

Model	Marigold petals		Guava leaves	
	pH 2.5	pH 6.5	pH 2.5	pH 6.5
Langmuir				
*q*_m_ (mg/g)	5.405	19.231	10.753	3.096
*b* (L/mg)	8.410	3.059	0.315	80.749
*R*^2^	0.519	0.077	0.327	0.826
Freundlich				
*K*_F_ (L/g)	103.234	29.904	2.765	4.208
*n*	1.112	1.206	1.074	3.831
*R*^2^	0.973	0.915	0.997	0.904
Temkin				
*K*_T_ (L/g)	215.649	159.309	31.248	43.921
*B*	1.116	1.609	0.448	0.284
*R*^2^	0.761	0.714	0.841	0.868
Dubinin–Radushkevich				
*q*_m_ (mg/g)	9.412	7.645	1.383	2.721
*E* (kJ/mol)	5.067	5.457	3.970	11.991
*R*^2^	0.953	0.887	0.939	0.897

The analysis of the data showed that the maximum adsorption capacity varies significantly according to the type of enterosorbent and the pH value (Table 4). Accordingly, marigold showed a much greater efficiency to remove AFB_1_ at the conditions of the stomach and intestine when using at doses of 0.25 and 0.125% (w/w), respectively. In fact, the Kf value of marigold was 37.3 and 7.1 times higher than those of guava leaves, respectively. Regarding the adsorption intensity (*n*), the empirical constant *n* indicates the degree of nonlinearity between the solution concentration and the adsorption as follows: if *n* < 1, the adsorption is a chemical process; if *n* = 1, the adsorption is linear; and if *n* > 1, the adsorption is a physical process. In the present research, the *n* value in the Freundlich model was found to be in the range of 1.074 to 3.831 (Table 4), indicative that adsorption of AFB_1_ onto the enterosorbents was dominated by physical mechanisms. Frequently, the case of *n* > 1 is very common during adsorption, and values within the range of 1–10 represent good adsorption (Özer and Pirincci 2006).

In this research, the Dubinin-Radushkevich isotherm model was also used to confirm the type of adsorption at the solid-water interface. Physisorption, ion exchange, and chemisorption frequently occur at *E* values of < 8, between 8 and 16, and > 20 kJ/mol, respectively. As shown in Table 4, the adsorption energy values calculated for marigold were 5.067 and 5.457 kJ/mol, indicating that adsorption of AFB_1_ in the conditions of the stomach and intestine is dominated by physical forces. In the case of the enterosorbent prepared from guava leaves, physical adsorption was also the governing mechanism for AFB_1_ uptake at the acidic conditions of the stomach (the *E* value was 3.970 kJ/mol). However, in the conditions of the intestinal section (pH 6.5), the energy value was 11.991 kJ/mol, indicating that ion exchange could also be involved during adsorption. Ion exchange could play an indirect role during adsorption specifically if plant-based materials contain charged components such as certain functional groups or minerals that can interact with the medium. In this work, guava leaves contained significant amounts of natural minerals such as calcium and magnesium (along with a considerable quantity of available silicates) embedded in their matrix as observed during the EDX analysis (Fig. 2d). Thus, ion exchange could involve these cations and counter-ions from the solution that mimics the intestinal section originating coordinated binding sites for AFB_1_ molecules. This phenomenon could help adsorption of the toxin indirectly onto the enterosorbent prepared from guava leaves by providing additional sites for π-π stacking or other interactions.

## Conclusion

This work assessed for the first time the potential of two enterosorbents to remove AFB_1_ under simulated gastric and small intestinal conditions. A preliminary screening demonstrated that marigold had an impressive removal efficiency at low inclusion rates at the conditions of the stomach and intestine. The adsorption process was dependent on the initial solution pH and the Freundlich isotherm was the most suitable among the four models in describing the experimental data. The *n* value in the Freundlich model was > 1, indicative that adsorption of AFB_1_ onto the enterosorbents was dominated by physical forces. Hydrogen bonding and hydrophobic interactions appear to be the major influencing factors driving AFB_1_ adsorption. Consequently, marigold petals and guava leaves could be considered promising binder materials to reduce the bioavailability of AFB_1_. However, future studies should focus on *in vivo* validation, long-term adsorbent stability, and nutrient interaction effects to provide a more comprehensive understanding of the applicability of these enterosorbents. Our laboratories are actively conducting research in this area.

## Supplementary Information

Below is the link to the electronic supplementary material.

ESM 1 (DOCX 473 KB)

## Data Availability

No datasets were generated or analysed during the current study.
