# Supplementary material for: Adsorptive potential of two natural enterosorbents for removing aflatoxin B1 under simulated gastric and small intestinal conditions
Source: Mycotoxin Res. 2025 Mar 20;41(2):373–83. doi: 10.1007/s12550-025-00588-z (PMC12037431; doi:10.1007/s12550-025-00588-z)
Supplement: Supplementary file 1 — (DOCX 473 KB) [file 12550_2025_588_MOESM1_ESM.docx]

(a)

(b)

(h)

(g)

(f)

(e)

(d)

(c)

**Fig. S1.** Adsorption isotherms of the enterosorbents prepared from marigold and guava leaves at the acidic condition of the stomach. (**a**, **b)** Langmuir; (**c**, **d)** Freundlich; (**e, f)** Temkin; and (**g, h)** Dubinin–Radushkevich, respectively.

(d)

(b)

(h)

(g)

(f)

(e)

(c)

(a)

**Fig. S2.** Adsorption isotherms of the enterosorbents prepared from marigold and guava leaves at the conditions of the small intestine. (**a**, **b)** Langmuir; (**c**, **d)** Freundlich; (**e, f)** Temkin; and (**g, h)** Dubinin–Radushkevich, respectively.
